# An Evaluation of the Text Illness Monitoring (TIM) Platform for COVID-19: Cross-sectional Online Survey of Public Health Users

**DOI:** 10.2196/32680

**Published:** 2022-02-07

**Authors:** Heather A Joseph, Susan Z Ingber, Chelsea Austin, Caroline Westnedge, F V Strona, Leslie Lee, Ami B Shah, Lauren Roper, Anita Patel

**Affiliations:** 1 Division of Environmental Health Science and Practice National Center for Environmental Health Centers for Disease Control and Prevention Chamblee, GA United States; 2 Division of Viral Hepatitis National Center for HIV, Viral Hepatitis, STD, and TB Prevention Centers for Disease Control and Prevention Atlanta, GA United States; 3 Department of Health Policy and Behavioral Sciences School of Public Health Georgia State University Atlanta, GA United States; 4 Federal Civilian Division General Dynamics Information Technology Atlanta, GA United States; 5 Division of STD Prevention National Center for HIV, Viral Hepatitis, STD, and TB Prevention Centers for Disease Control and Prevention Atlanta, GA United States; 6 Office of the Director National Center for Immunization and Respiratory Diseases Centers for Disease Control and Prevention Atlanta, GA United States

**Keywords:** COVID-19, contact tracing, SMS text system, symptom monitoring

## Abstract

**Background:**

The US public health response to the COVID-19 pandemic has required contact tracing and symptom monitoring at an unprecedented scale. The US Centers for Disease Control and Prevention and several partners created the Text Illness Monitoring (TIM) platform in 2015 to assist US public health jurisdictions with symptom monitoring for potential novel influenza virus outbreaks. Since May 2020, 142 federal, state, and local public health agencies have deployed TIM for COVID-19 symptom monitoring.

**Objective:**

The aim of this study was to evaluate the utility, benefits, and challenges of TIM to help guide decision-making for improvements and expansion to support future public health emergency response efforts.

**Methods:**

We conducted a brief online survey of previous and current TIM administrative users (admin users) from November 28 through December 21, 2020. Closed- and open-ended questions inquired about the onboarding process, decision to use TIM, groups monitored with TIM, comparison of TIM to other symptom monitoring systems, technical challenges and satisfaction with TIM, and user support. A total of 1479 admin users were invited to participate.

**Results:**

A total of 97 admin users from 43 agencies responded to the survey. Most admin users represented the Indian Health Service (35/97, 36%), state health departments (26/97, 27%), and local or county health departments (18/97, 19%), and almost all were current users of TIM (85/94, 90%). Among the 43 agencies represented, 11 (26%) used TIM for monitoring staff exclusively, 13 (30%) monitored community members exclusively, and 19 (44%) monitored both staff and community members. Agencies most frequently used TIM to monitor symptom development in contacts of cases among community members (28/43, 65%), followed by symptom development among staff (27/43, 63%) and among staff contacts of cases (24/43, 56%). Agencies also reported using TIM to monitor patients with COVID-19 for the worsening of symptoms among staff (21/43, 49%) and community members (18/43, 42%). When asked to compare TIM to previous monitoring systems, 78% (40/51) of respondents rated TIM more favorably than their previous monitoring system, 20% (10/51) said there was no difference, and 2% (1/51) rated the previous monitoring system more favorably than TIM. Most respondents found TIM favorable in terms of time burden, staff burden, timeliness of the data, and the ability to monitor large population sizes. TIM compared negatively to other systems in terms of effort to enroll participants (ie, persons TIM monitors) and accuracy of the data. Most respondents (76/85, 89%) reported that they would highly or somewhat recommend TIM to others for symptom monitoring.

**Conclusions:**

This evaluation of TIM showed that agencies used TIM for a variety of purposes and rated TIM favorably compared to previously used monitoring systems. We also identified opportunities to improve TIM; for example, enhancing the flexibility of alert deliveries would better meet admin users’ varying needs. We also suggest continuous program evaluation practices to assess and respond to implementation gaps.

## Introduction

Monitoring exposed individuals during a public health crisis, such as the COVID-19 pandemic, is critical for implementation of an effective public health response. Ongoing symptom monitoring conducted by clinical providers and public health officials has traditionally been done via telephone calls or in-person screening, both time-consuming processes requiring extensive health department resources. Text-based communication is increasingly used for public health interventions [1-3]. Two-way SMS text messaging can scale up public health agencies’ ability to monitor on a predetermined schedule (ie, daily or weekly) or conduct a one-time follow-up with individuals.

The US Centers for Disease Control and Prevention (CDC) has recommended the use of technology to enhance partner services for sexually transmitted infections since 2008 [4]. For example, CDC’s Toolkit for Technology-Based Partnership Services provides guidance on using text messaging and mobile apps to help providers initiate appointment setup for partners of infected patients, as well as patient check-in and monitoring medication compliance [5]. Text platforms have also been used for monitoring postpartum depression and mental health [6,7], symptom monitoring for infectious disease contact tracing during Ebola [8], and symptom monitoring and prophylaxis medication adherence for avian influenza and influenza-like illness [9,10]. In sum, the use of text-based monitoring systems continues to grow, and preliminary reports are encouraging regarding their usability, acceptability, and effectiveness in an acute infectious disease outbreak. However, because the aims, populations monitored, and platforms vary greatly, it is critical to conduct evaluation to improve delivery and build the evidence base for emergent needs and systems.

In 2020, CDC recommended that those potentially exposed to SARS-CoV-2 be monitored for 14 days [11]. This posed a burden to public health systems, which could have led to substantial under monitoring. In the context of the COVID-19 pandemic, the World Health Organization has encouraged the use of electronic data capture tools to support efficient contact tracing and active surveillance of close contacts on a large scale [12]. Though evidence for the effectiveness of text-based active surveillance or monitoring systems among community-based contacts of cases of COVID-19 has been limited to date, several reports indicate some promise [13-16].

The Text Illness Monitoring (TIM) platform was developed in 2015, through a collaboration between CDC, the National Association of County and City Health Officials, and Compliant Campaign (a third-party contractor) to assist US jurisdictions with monitoring individuals at potential risk for novel influenza virus infection. In 2016, the Michigan Department of Health and Human Services asked CDC to pilot-test TIM during a swine flu outbreak at nine county fairs [17]. The pilot evaluated TIM’s ability to enhance detections of H3N2v virus infections among household members of symptomatic fair attendees and its feasibility and acceptability for use in future outbreak investigations of novel influenza viruses or similar threats. Among an estimated 500 households with a member who exhibited symptoms, representatives of 87 (17.4%) households were enrolled in TIM. Ultimately, the system detected two H3N2v virus infections among the enrolled household members, and 80% of survey respondents indicated they would participate in another TIM-based monitoring event.

Early in the COVID-19 pandemic, there was no existing system that was free, rapidly available, and easy to scale up for symptom monitoring of large, diverse populations. At the time, public health entities, like state and local health departments, were overwhelmed with trying to identify broader control actions, conduct surveillance, organize laboratory activities, and prepare the nation for the response to the pandemic. As a result, CDC reconfigured TIM to meet these pressing challenges of symptom monitoring for large-scale contact tracing and employee monitoring, while promoting and providing technical support for TIM to domestic public health agencies at no cost. CDC began to use TIM to monitor deployed staff in February 2020, as a pilot. Shortly thereafter, multiple federal agencies began using TIM to monitor symptoms among employees. State, local, and tribal public health authorities quickly followed by using TIM to monitor staff and community members for development and worsening of symptoms. Use of TIM was voluntary for federal agencies and public health authorities. Over the spring and summer of 2020, CDC continued to expand monitoring of field-deployed and remotely deployed responders; CDC staff administering TIM provided regular and detailed feedback to the development team to support enhancements.

In response to heightened interest in TIM, ad hoc user feedback, and suggestions for improving the system, CDC initiated an evaluation of the TIM system in accordance with CDC’s Framework for Program Evaluation to facilitate selection and prioritization of system enhancements [18]. The evaluation team convened internal stakeholders who established the following evaluation questions: Who are the administrative users (admin users)? What are the most important reasons for adopting TIM? How is TIM being used by public health agencies? How does TIM compare to other monitoring systems? To what extent has TIM contributed to earlier COVID-19 identification? What are the challenges of using TIM and how can these be addressed? How satisfied are admin users with TIM and CDC support?

For the purpose of better understanding the needs of admin users, we also wanted to discern if there were differences in these outcomes by the type of agency or the populations being monitored.

## Methods

### Text Illness Monitoring Platform

**Figure 1:**
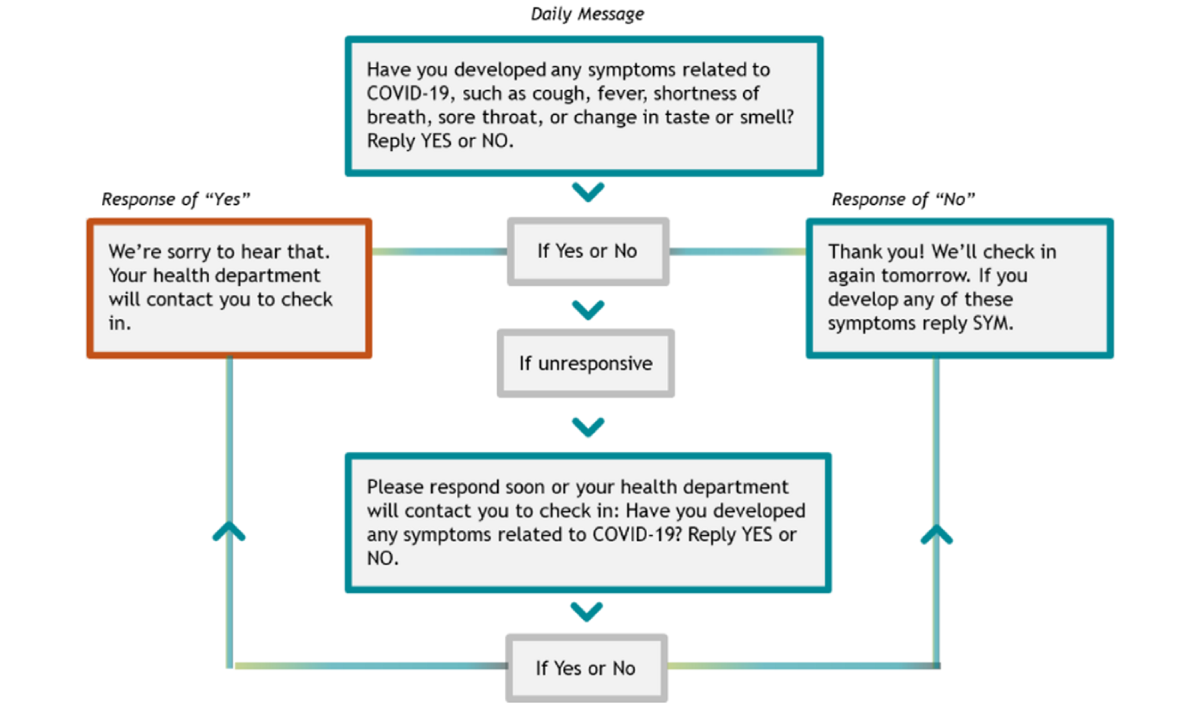
Daily message from a typical Text Illness Monitoring campaign for COVID-19.

TIM was designed to be a simple, low-resource tool to implement. In the context of the current use for the COVID-19 response in the United States, the CDC TIM team enrolls public health officials (ie, “admin users”), who can then create “campaigns”—text messaging workflows in which participants are enrolled for monitoring—for their jurisdictions. A typical campaign workflow for the daily message is shown in Figure 1.

**Figure 2:**
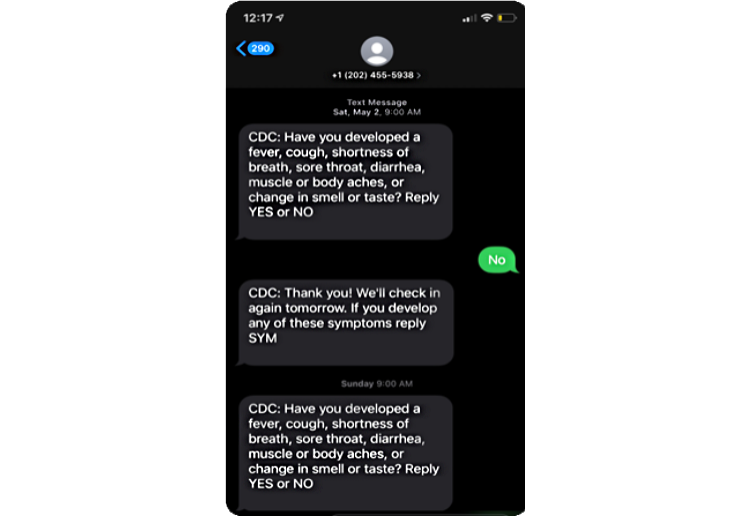
Example text exchange on the Text Illness Monitoring platform for COVID-19.

Persons whom TIM monitors for symptoms (ie, “participants”) receive two or more text messages each day for the monitoring period designated by the admin user. As shown in Figure 2, texts to participants include questions about whether they have symptoms consistent with COVID-19 and require a “YES" or "NO” response. TIM generates an alert when a participant responds “YES” or “SYM” (ie, symptom). TIM can read other response variations (ie, “Y,” “Yea,” or “symptom”) that confirm experiencing symptoms and generates a symptom alert. If a participant does not respond to the first message by a preset time, TIM sends a follow-up reminder. If the participant does not respond by a preset time to the reminder text, TIM will create a nonresponse alert. A dashboard feature allows TIM admin users to log in, track, and follow up with participants who report symptoms or fail to respond. Admin users can view alerts outside of the dashboard if they opt to receive email notifications for alerts. Participants can opt out of TIM at any time by texting “QUIT,” “END,” “CANCEL,” “UNSUBSCRIBE,” “REMOVE,” “OPT OUT,” “OPTOUT,” or “STOP.” Admin users can also stop messages at any time or can set a specific number of days for the monitoring period via the dashboard. If a participant sends an unexpected response to TIM, the system sends a standard response to prompt the participant to send an appropriate response (eg, “You are enrolled in the text symptom monitoring program. If you develop symptoms, reply SYM.”).

Reflecting the capability for local tailoring, admin users can customize the technical attributes of their TIM campaigns (eg, number of monitoring days, language selection, and time limit between initial and reminder messages). TIM allows for multiple admin users with tiers of access to control interactions for various user levels that were specifically designed to manage state-level versus local-level views for the system.

Between May 1 and December 29, 2020, a total of 682 TIM campaigns were established. This included 1479 staff from 142 public health agencies in 20 states who were designated as TIM admin users, and 97,184 individuals across the United States who were enrolled as TIM participants. The daily average number of persons monitored was 6838. For the week of November 28 to December 4, 2020 (ie, the first week of data collection for this evaluation), the weekly average of participants reporting symptoms was 3.8%, and the weekly average nonresponse to alerts was 17.4%.

### Survey Design

To validate the instrument, we piloted survey questions through seven semistructured, open-ended, 60-minute phone interviews with a sample of TIM admin users. Eligible respondents for the validation phase and the online survey were TIM admin users associated with a current or formerly active campaign established at least 2 weeks prior to the interview or survey completion. We conducted phone interviews with 7 admin users who had a wide range of experiences with TIM, based on administrative data from the TIM Support Team (see Multimedia Appendix 1 for interview instrument). We incorporated insights from the validation stage into the survey (Multimedia Appendix 2) that was deployed to admin users via REDCap (Research Electronic Data Capture). REDCap is a secure, Health Insurance Portability and Accountability Act (HIPAA)–compliant, web-based application developed by Vanderbilt University and used by CDC to capture research data and create databases.

### Data Collection

The TIM Support Team emailed a survey invitation to all admin users (n=1479). The anonymous survey took approximately 15 minutes to complete and, as indicated on the introduction page, multiple admin users per agency were eligible to respond. No incentives for survey completion were provided. Respondents indicated their state and agency name. The survey invited respondents to answer questions, all optional, about the following topics: onboarding process, decision to use TIM, use of TIM-generated data and reports, technical challenges of using TIM, utility of TIM, and satisfaction with TIM and user support. The survey also included two questions that prompted respondents to report the following for their agency: populations monitored and uses of TIM. A respondent may have worked for an agency with multiple campaigns; we sought to collect this information about all of the campaigns for a particular agency. Survey question types included “check all that apply,” yes-or-no, multiple-choice, Likert-scale, and open-ended questions to collect both quantitative and qualitative feedback. For example, we asked respondents to compare TIM to the most recent system used prior to TIM through a series of questions about cost, staffing requirements, staff hours, burden to enroll participants, number of participants that could be monitored, data accuracy, data completeness, and data timeliness. Participants rated these on a 5-point scale with a neutral response option in the middle (ie, “the same”). We categorized “better” and “somewhat better” responses as “favorable,” and we categorized “somewhat worse” and “much worse” responses as “unfavorable.” The data collection period ran from November 28 to December 21, 2020. A copy of the survey questions is presented as Multimedia Appendix 2.

### Analysis

The quantitative data were analyzed in SAS 9.4 (SAS Institute Inc) using standard analysis techniques, including basic descriptive statistics. The primary unit of analysis is the respondent (ie, admin user). For the analysis on the use of TIM, we report the agency as the unit of analysis because the use of TIM is an agency-level decision. We also sought to discern if there were differences by type of agency and populations monitored, as this information could be used to refine the application to better meet admin users’ needs. Due to low sample sizes, crude frequencies and the Fisher exact test were used to determine statistical differences in terms of agency type or population monitored. We report the results of this statistical test when differences were statistically significant (*P*<.05). Since all questions were optional, there were missing data. Consequently, denominators vary by question.

Qualitative data collected via open-ended questions were input into a separate data set for coding. Two members of the research team used an inductive approach to develop an initial set of codes on a subsample of the extracted qualitative data (ie, approximately 10% of all extracted content) [19]. Both coders then independently coded samples of data and met twice weekly to reconcile coded content and update their code list and definitions [20]. This process was repeated until intercoder agreement reached 84% [19,21]. From there, a single coder finished coding the remaining qualitative data (ie, approximately one-third of the total sample). Both coders then performed content analysis to determine those themes that emerged most prominently [22]. To enable a better understanding of theme salience, coders quantified the frequency of theme mentions across the data set, by number of survey respondents and by number of unique agencies. We also assessed whether patterns may vary by agency type.

### Compliance

CDC determined that the data collection was nonresearch; thus, no human subjects review was conducted in accordance with applicable federal law and CDC policy. The Paperwork Reduction Act applied, but a public health emergency waiver was obtained.

Regarding informed consent, the survey home page included introductory information about the reason for the data collection, voluntary participation, nonidentifiable reporting of the findings, length of time for survey completion, and whom to contact about TIM or the survey. There was no documentation of consent.

## Results

### Response Rate

During the time of data collection from November 28 through December 21, 2020, 67 agencies in 18 states were actively using TIM. A total of 180 monitoring campaigns were in progress, and 10,414 participants were being monitored.

Of 1479 admin users contacted to respond to the survey, 100 (6.8%) responded. Out of those 100 respondents, 2 (2.0%) were ineligible because they had not established a campaign. We also found 1 (1.0%) incomplete duplicate of a complete survey. These 3 (3.0%) records were dropped, resulting in an analytic sample of 97 respondents, representing 43 distinct agencies. Out of 97 respondents, 11 (11%) did not specify their agency name. Specifically, 2 respondents from Florida were assigned to a single “Florida_Unspecified” group and 9 Indian Health Service (IHS) respondents were assigned to a single “IHS_Unspecified” group.

The response rate for individual admin users was low (100/1479, 6.8%), which could have been the result of many users never accessing or using the system or due to the frequent turnover of public health staff at the local level. We could not retrospectively identify “active admin users” at a given time through the administrative data provided by the platform developer. However, we were able to determine an agency-level response rate, which was higher. Staff from 30.3% (43/142) of agencies that had ever used TIM responded, while staff from 64% (43/67) of agencies that were actively using TIM at the time of the data collection responded to the survey.

### Respondent Characteristics

Respondents represented a diverse sample of public health agencies (Table 1). Almost all respondents were current admin users of TIM, and slightly over one-third reported using the system for 3 months or less. The mean number of months using TIM was 4.5 (SD 2.55), with a range of less than 1 month to 10 months. Though only 39% (37/94) of respondents indicated that they were primary or secondary points of contact for TIM within their agency, most were responsible for managing one or more features of TIM (eg, administration of campaigns, participants, alerts, user features, and data and reporting features).

**Table 1:** Characteristics of respondents of the online survey regarding the Text Illness Monitoring (TIM) platform for COVID-19, November to December 2020.

Characteristic			Respondents (N=97), n (%)
**Agency type^a^** **(N=97)^b^**			
	Indian Health Service	35 (36)	
	State	26 (27)	
	Local or county	18 (19)	
	Federal (other than Indian Health Service)	12 (12)	
	Tribal nation	6 (6)	
Currently using TIM (n=94)			85 (90)
**Number of months using TIM (n=93)**			
	<1-3	36 (39)	
	4-6	34 (37)	
	≥7	23 (25)	
**Role (n=94)**			
	Primary or secondary point of contact for TIM	37 (39)	
	Responsible for managing one or more features of TIM	89 (95)	

^a^To simplify subsequent analysis for “agency type,” state and local agencies were combined into one category, and tribal nation agencies and the Indian Health Service were combined into another.

^b^Denominators vary because of nonresponse, in the form of missing responses and responses of “I don’t know.”

### Reasons for Adopting TIM

As shown in Table 2, respondents indicated that the most common reasons for adoption were the ability to monitor large populations and that TIM is a better alternative to phone screening. Compared to respondents from state and local agencies, those from the IHS and tribal nation agencies more frequently indicated that the rationale for adoption was that TIM was a better alternative to in-person screening (20/40, 50%, vs 9/40, 23%; *P*=.02).

**Table 2:** Key survey results regarding evaluation of the Text Illness Monitoring (TIM) platform for COVID-19, November to December 2020.

Survey item and responses				Value, n (%)
**Respondent level^a^**				
	**Reasons for adopting TIM (n=90)^b^**			
		Could monitor large numbers of people	53 (59)	
		Better alternative to screening via phone	42 (47)	
		Better alternative to screening in person	34 (38)	
		No cost	27 (30)	
		Created and maintained by US Centers for Disease Control and Prevention	19 (21)	
	**Systems previous to TIM**			
		Used a system before TIM (n=90)	64 (71)	
		Used in-person screening (n=63)	31 (49)	
		Used other contact tracing software or symptom monitoring application (n=63)	11 (17)	
	**Identification of COVID-19 symptoms (n=87) and confirmed cases (n=86)**			
		TIM identified symptomatic participants (yes)	70 (80)	
		TIM identified confirmed cases (yes)	44 (51)	
	**Reported that TIM identified symptomatic participants in a timely way (n=69)**			
		Somewhat	25 (36)	
		A lot	30 (43)	
		Very much so	14 (20)	
	**Satisfied or very satisfied with the TIM Technical Support Team (n=26)**			
		Timeliness	25 (96)	
		Communication	24 (92)	
		Extent to which issues were resolved	22 (85)	
	**Would recommend TIM for symptom activity monitoring (n=85)**			
		Highly or somewhat recommend	76 (89)	
		Neither recommend nor discourage	6 (7)	
		Highly or somewhat discourage	3 (4)	
	**Other tools used alongside TIM (n=92)**			
		Spreadsheets	64 (70)	
		Agency or personal phones	43 (47)	
		In-person screening	30 (33)	
		Pen and paper	29 (32)	
		Contact tracing software	19 (21)	
		Data analysis software	5 (5)	
		None	5 (5)	
**Agency level^c^**				
	**Populations monitored (n=43)**			
		Monitoring staff only	11 (26)	
		Monitoring community members only	13 (30)	
		Monitoring both staff and community members	19 (44)	
	**Uses of TIM (n=43)**			
		Among staff, monitoring for development of symptoms	27 (63)	
		Among staff, monitoring cases for worsening of symptoms	21 (49)	
		Among staff, monitoring contacts of cases for development of symptoms	24 (56)	
		Among community members, monitoring cases for development of symptoms	20 (47)	
		Among community members, monitoring cases for worsening of symptoms	18 (42)	
		Among community members, monitoring contacts of cases for development of symptoms	28 (65)	

^a^Questions were asked in a way that indicated respondents could answer for themselves or their agency.

^b^Denominators vary because of nonresponse, in the form of missing responses and responses of “I don’t know.”

^c^Questions were asked in a way that indicated respondents should answer for their agency.

### Uses of TIM

We report uses of TIM by agency, rather than by individual respondent (Table 2). We found a mix of uses and populations monitored. In terms of populations monitored, agencies reported using TIM to exclusively monitor illness in staff, community members, or both groups.

Agencies most frequently used TIM to monitor for symptom development in contacts of cases among community members, followed by symptom development among staff and among staff contacts of cases. Agencies also reported using TIM to monitor COVID-19 cases for the worsening of symptoms among staff and community members. Another reported use was for monitoring symptom development in contacts of cases among community members.

Federal agencies (3/3, 100%) and IHS and tribal nation agencies (12/19, 63%) most frequently used TIM to monitor for development of symptoms among staff, while state and local agencies (18/21, 86%) most frequently used TIM to monitor community contacts of cases for development of symptoms.

In conjunction with using TIM for symptom monitoring, agencies reported using spreadsheets, agency or personal phones, in-person screening, pen and paper, contact tracing software, data analysis software, and no other tools.

### Comparison of TIM to Previously Used Monitoring Systems

**Figure 3:**
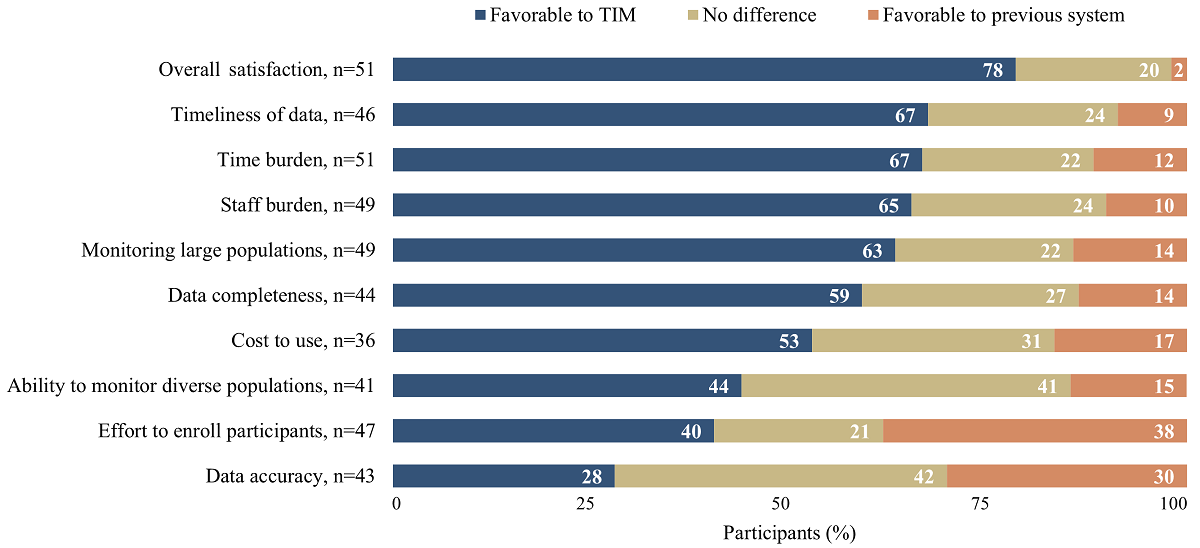
Evaluation of the Text Illness Monitoring (TIM) platform for COVID-19, comparison of TIM to previous monitoring system, November to December 2020.

Most respondents reported using another monitoring system before TIM. These systems included in-person screening and other contact tracing software or symptom monitoring applications (Table 2). As shown in Figure 3, among those who had used a previous system, most rated TIM favorably overall compared to the previous system they used; 11 respondents indicated “I don’t know” to all questions. TIM compared most favorably to the previous system in terms of the timeliness of the data, time burden, staff burden, and the ability to monitor large population sizes. Compared to TIM, respondents indicated that their previous systems required less effort to enroll participants and yielded more accurate data.

Compared to IHS and tribal nation agencies, state and local agencies were more likely to consider TIM to be favorable for monitoring larger populations (19/23, 83%, vs 10/23, 43%; *P*=.01).

### Identification of COVID-19 Symptoms and Confirmed Cases

As shown in Table 2, most respondents reported that TIM identified participants who developed symptoms of COVID-19 and participants who were later confirmed to be cases. Respondents indicated that TIM identified symptomatic participants in a timely way either “very much so,” “a lot,” or “somewhat.”

### Challenges of Using TIM

**Figure 4:**
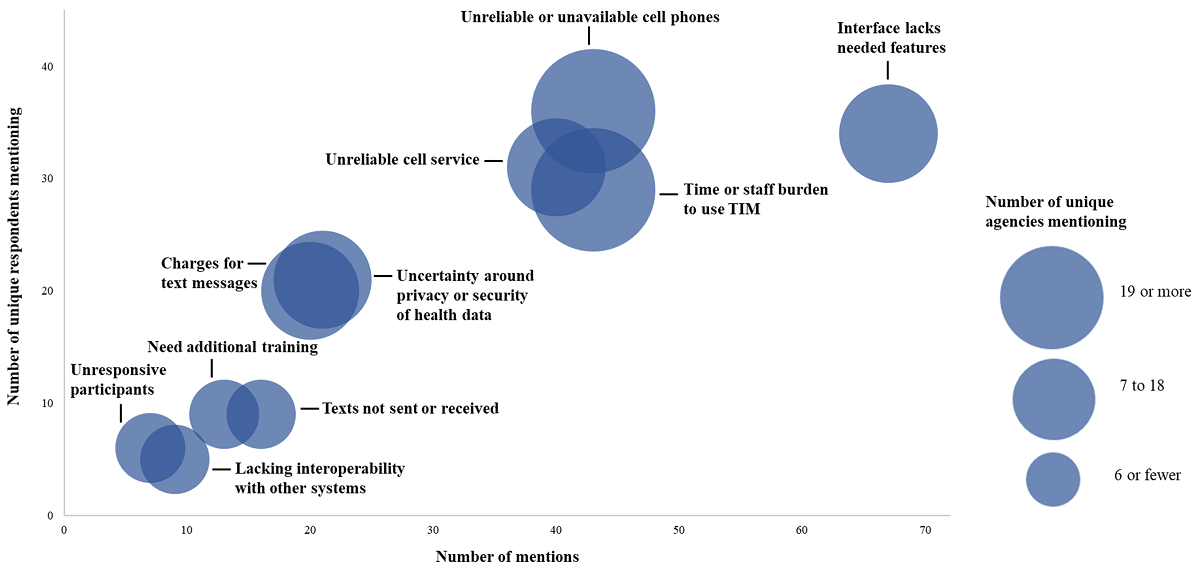
Evaluation of the Text Illness Monitoring (TIM) platform for COVID-19, reported challenges using TIM, November to December 2020. Content analysis themes were mapped according to their number of mentions (x-axis), the number of unique respondents (y-axis), and the number of agencies reporting the challenge (bubble size). Thus, larger bubbles placed further along the x-axis and y-axis indicate more prevalent challenges.

Respondents were asked a series of open-ended questions about technical challenges and concerns regarding the use of TIM. Figure 4 shows the prominent themes that emerged during content analysis. The most prevalent issues reported included lack of needed features in the TIM interface (67 mentions among 34 respondents), staff or time burden (43 mentions among 29 respondents), and unreliable cell phones or service, which could sometimes be altogether unavailable (43 mentions among 36 respondents, and 40 mentions among 31 respondents, respectively).

The most requested feature enhancements were the ability to customize campaign time zones, to receive symptom alerts by email or text message, and to delete or move participants from campaigns at any time. Participants reported time or staff burden accruing through efforts to enroll large numbers of participants and difficulty sorting or navigating through many pages of data generated by nonresponse alerts. Of the unique agencies reporting “unreliable cell service” as a concern, a little over half (10/17, 59%) were IHS or tribal nation affiliated. Additionally, of the unique agencies reporting “unreliable or unavailable cell phones,” well over half (12/19, 63%) were IHS or tribal nation affiliated.

Some themes co-occurred across the data set, suggesting underlying relationships among reported challenges. Slightly less than half (19/43, 44%) of the “time or staff burden” mentions co-occurred with “interface lacks needed features” mentions. Additionally, nearly half (7/16, 44%) of the “texts not received or sent” mentions co-occurred with other themes, namely unreliable cell service (4/7, 57%) and unreliable or unavailable cell phones (2/7, 29%).

### Satisfaction With CDC Support and Overall Experience

CDC provided onboarding support that included sending welcome emails to introduce TIM, conducting TIM orientations and demonstrations, and providing the TIM user guide and frequently asked questions (FAQs). CDC also staffed a help desk to provide daily support for admin users after they established their campaigns. Among those who submitted technical support requests to the TIM Support Team, most respondents indicated that they were satisfied with the timeliness of the TIM Support Team’s responses to their tech support requests (Table 2).

The survey also collected data about whether respondents would recommend TIM to others for symptom monitoring. Most respondents (76/85, 89%) reported that they would highly or somewhat recommend TIM, while a few were neutral (6/85, 7%) or would discourage others from using TIM (3/85, 4%). This did not vary by agency type or population monitored.

## Discussion

### Principal Findings

Evaluation of emergency public health response activities can provide timely and actionable insights about successes and areas for improvement to responders and decision makers. In the context of the COVID-19 response, CDC has encouraged state and local health departments to implement and use evaluation findings on topics ranging from mask wearing to COVID-19 mitigation strategies in schools. With this same intention, this evaluation of TIM can help support its expanded use for the current COVID-19 pandemic and provide guidance for TIM use during future public health emergencies. The evaluation also provides evidence for prioritizing specific system enhancements to better support TIM admin users with implementation.

In sum, admin users who responded to the survey represented the IHS, state health departments, and local or county health departments. Agencies represented by survey respondents used TIM to exclusively monitor staff, monitor community members, or both. Admin users’ agencies most frequently used TIM to monitor symptom development in contacts of cases among community members, followed by symptom development among staff and among staff contacts of cases. Agencies also used TIM to monitor patients with COVID-19 for the worsening of symptoms among staff and community members. Most respondents rated TIM more favorably compared to their previous monitoring system. Most respondents found TIM favorable in terms of time burden, staff burden, timeliness of the data, and the ability to monitor large population sizes. TIM compared negatively to other systems in terms of effort to enroll participants and accuracy of the data. Most would highly or somewhat recommend TIM to others for symptom monitoring.

TIM is intended to aid in the timely detection, treatment, and prevention of transmission of viruses with pandemic potential, such as COVID-19. Passive surveillance methods can miss infections, while other active surveillance strategies, like in-person or phone screening, can be very time- and resource-intensive. This evaluation supports the results of the initial study of TIM’s use during a swine flu outbreak at agricultural fairs [17]. In that use case, TIM successfully identified two cases among the 392 individuals monitored for illness over a 4-week period. Stewart et al [17] reported that two types of text messages were sent: one using formal language and another using informal language. The informal version was associated with more staff follow-up time due to false alerts and unrecognized text responses. The version of TIM deployed for the COVID-19 response provided admin users with the flexibility to customize the level of formality for their TIM campaigns, but also defaulted the system to use more formal and straightforward language to avoid similar monitoring challenges as those experienced during the swine flu outbreak. No survey respondents in this evaluation indicated that participants had difficulty understanding the expected text response.

SMS text-based systems like TIM are important tools for large outbreak investigations that require significant public health resources, as they are not only scalable but also cost-effective. These systems are easier to use than mobile apps, which may require downloading, favor more technologically savvy admin users, and can invoke privacy concerns [23]. Anecdotally, new admin users typically created a campaign and began enrolling participants into TIM within 1 to 2 weeks of gaining access to the platform (personal communication, CW). Survey respondents often reported uses for both community members and staff, reflecting TIM’s flexibility; in fact, some admin user agencies implemented multiple concurrent campaigns for different types of participants. Agencies used TIM to monitor participants for the development of symptoms, as well as the worsening of symptoms among confirmed COVID-19 cases. Often used with other support tools and activities, TIM was also integrated into other program outbreak response operations, such as contact tracing.

According to respondents, TIM not only successfully identified symptomatic participants—some of whom were later identified as confirmed COVID-19 cases—but also did so in a timely manner. The survey results indicated that admin users were generally satisfied with TIM, comparing it favorably to previous systems used, especially in terms of cost, timeliness of data, data completeness, staff burden, time burden, and ability to monitor large populations. This parallels the evaluation findings of an Australian SMS text messaging program administered during a 2013 poultry farm outbreak of avian influenza to monitor for symptom development among exposed individuals. The study found that monitoring via SMS text messages was less time-consuming and more cost-effective than conducting telephone follow-up [9]. TIM was rated less favorably in terms of effort to enroll participants and data accuracy. We did not define the term “accuracy” in the survey instrument. However, the pilot interviews reflected that some admin users were concerned that monitored participants may be less likely to accurately report their symptoms via text compared to speaking to monitoring staff via phone. Given that survey respondents may have interpreted the term “accuracy” differently, we are unable to make a conclusion. This could be explored and verified with current TIM admin users.

TIM is similar to other COVID-19 text-based systems in that resources and staff time are required for monitoring daily reports for follow-up, oversight, and data protection safeguards [14,15]. Our qualitative findings highlighted respondents’ particular challenges with TIM, including lack of interface features and cell phone and service access. CDC’s implementation of the most feasible requested interface enhancements to TIM may alleviate some of the staff time burden. Since the majority of respondents who mentioned “texts not received or sent” also reported cell phone and service access issues, message interruptions likely came from outside of the TIM platform.

Some respondents indicated needing to set up multiple systems to ensure 100% capture (eg, using phone calls to augment texts). While this may be required for full participation, direct texting may be the most desirable mechanism for most participants. An evaluation of a COVID-19 text message system used in Maine, United States, found that the majority of participants who agreed to be monitored via an automated system preferred direct text (60.2%) versus texted weblink (21.15%), telephone call (7.8%), or emailed weblink (7.3%) [15]. The vast majority (89%) of admin user respondents for this evaluation indicated their overall satisfaction with TIM and reported that they would recommend TIM for managing symptom monitoring activities.

The survey also collected data about admin user experience with technical support issues and requests. The CDC TIM Support Team is composed of one full-time technical lead, one full-time technical coordinator, one 50%-time data analyst, and one 25%-time senior lead, along with the application contractor. Most of the team consisted of Emergency Operation Center responder staff, which has a high turnover requiring regular training. The team provided orientations and onboarding assistance for new, potential admin users; updated and shared training materials; hosted TIM question-and-answer sessions; and provided daily responses to technical support requests. Respondents were overwhelmingly satisfied with the support provided by the TIM Support Team as well as the documented guidance provided for onboarding. This illustrates the essential nature of support staff when developing SMS text monitoring services meant to be used by diverse audiences.

### Recommendations

Based on these findings, we offer several recommendations for the TIM platform and others considering the use of SMS-based tools for symptom monitoring. First, providing alternative delivery mechanisms to the dashboard, such as text messages for viewing symptom alerts, may improve user experience by eliminating the need for admin users to log in to TIM to view alerts data on the TIM dashboard. Although email alerts are supported by TIM, this option is limited to allowing only one admin user to be able to receive symptom and nonresponse alerts.

Second, the promotion of TIM to other potential admin users could be improved by highlighting the comparative advantages of TIM reported by the survey respondents. Additionally, CDC can clarify the current limitations of TIM’s customizability and provide guidance during the admin user onboarding process for assessing the appropriateness of this tool for certain populations. For example, administrators in rural areas with poor cellular coverage, or where populations lack mobile phones, may want to consider deploying an alternative or complementary system that addresses these circumstances. In some public health emergencies, it may be warranted to provide mobile phones to individuals who lack such devices and only require symptom monitoring for a limited period of time [24]. On a larger scale, the value of rural infrastructure that includes expanded mobile service is highlighted by the necessary capacity of public health authorities to conduct routine public health contact tracing and symptom monitoring [25].

Future evaluations of TIM could include a comparison of quantitative metrics reflecting retention, accuracy, and cost between TIM and the alternative and existing systems based on phone screening or in-person visits. We also suggest adopting continuous program evaluation practices to assess and respond to implementation gaps. Future evaluations could also be expanded to include participants’ experience with TIM. Additionally, the TIM team can use administrative data to flag issues in real time. For example, multiple nonresponse alerts can indicate that a participant is not receiving texts. Additionally, the TIM team can categorize and tag help-desk tickets to prioritize potential areas for added functionality or training. Along similar lines, this evaluation and others [16] highlight the importance of engaging with primary and secondary users of tools such as TIM to validate assumptions and understand user perspectives. Last, the cohort of TIM admin users who are no longer using the system may also represent an important audience from which to collect feedback on user experience and reasons for discontinuation. While this study did attempt to recruit this population, few previous admin users responded; therefore, targeted recruitment efforts are likely required.

### Limitations

This evaluation has some notable limitations. Though the survey response rate was higher in terms of agency-level representation, the low response rate (6.8%) among all current and previous TIM admin users potentially introduces bias because survey respondents may not share the same opinions as nonrespondents. The low response rate may have been due to the high work demands and high turnover among staff working on the COVID-19 response during the first peak event combined with the holiday season. Additionally, multiple admin users per agency were eligible to respond to the survey, which could introduce sampling bias. However, an agency may have had multiple, ongoing campaigns, each of which may have been managed by a different TIM user at the agency. The data presented in this manuscript are not meant to be generalizable to all past or current TIM admin users. Also, the survey’s cross-sectional design and single data collection point precluded analysis across time points in the COVID-19 pandemic.

The survey was shared with all current and previous TIM admin users and, therefore, had to accommodate varying levels of familiarity and expertise for TIM implementation. Consequently, a large proportion of respondents (up to 36%) reported “I don’t know” responses to the questions that invited comparisons of TIM to previously used systems and were removed from that analysis. It is unclear why so many participants responded this way to these questions, but some potential reasons include being responsible for limited aspects of TIM administrative activities; not being trained in, or not having experience with, specific features of the system; and lack of knowledge about the previous or alternative systems that were used.

Most respondents were the single survey respondent for an agency (n=27). Most other instances of multiple respondents from an agency were limited to 2, 3, 4, 5, and 6 respondents (n=9, 1, 1, 1, and 1, respectively). However, there were 3 agencies that had high representation: Florida Health Department (n=17), “IHS_Unspecified” (n=9), and CDC (n=9). Because Florida manages county-level health departments at the state level, we suspect that respondents from the Florida Health Department who reported state affiliations actually work at the county level. Out of 9 respondents representing “IHS_Unspecified,” 8 were from four different regions. Nevertheless, there is potential for respondents from the same agency to be similar and, thus, bias the results. We were unable to conduct intraclass correlation due to the limited sample size.

Additionally, CDC was unable to obtain several important administrative data elements that would have helped tell a more complete story about symptom and nonresponse alert rates, since the TIM admin user agreement precluded CDC access to these data. TIM’s allowance for episodic use (ie, allowing admin users to start and stop use at any time) meant that many enrolled admin users had the option to monitor symptoms for periods of time as short as days or weeks, potentially going on to never use the platform again. Consequently, some of the outcomes under study (eg, comparisons to other systems and identification of COVID-19 cases) may have been difficult to realize or observe for some admin users.

### Implications and Conclusions

TIM could continue to play a valuable role in state and local health departments’ COVID-19 responses when case numbers decrease and when more intensive contact tracing efforts resume to bring transmission rates to zero. TIM could potentially be used for post–COVID-19 vaccination safety monitoring. It can also be applied to other large-scale infectious disease outbreaks that feature a finite period for symptom monitoring, such as avian influenza. Other possible use cases for TIM beyond infectious disease outbreak response include monitoring of adverse reactions to vaccination, medication adherence, and health and symptoms of persons with certain chronic diseases or substance use disorders. While the potential uses of tools such as TIM are limitless, the role of evaluation to understand the user experience will remain essential for ensuring their successful implementation.

## Appendix

Multimedia Appendix 1 Interview instrument.

Multimedia Appendix 2 Survey instrument.

## Abbreviations

admin user: administrative user
CDC: Centers for Disease Control and Prevention
FAQs: frequently asked questions
HIPAA: Health Insurance Portability and Accountability Act
IHS: Indian Health Service
REDCap: Research Electronic Data Capture
TIM: Text Illness Monitoring
