# Supplementary material for: An Evaluation of the Text Illness Monitoring (TIM) Platform for COVID-19: Cross-sectional Online Survey of Public Health Users
Source: JMIR Public Health Surveill. 2022 Feb 7;8(2):e32680. doi: 10.2196/32680 (PMC8823610; doi:10.2196/32680)
Supplement: Multimedia Appendix 1 [file publichealth_v8i2e32680_app1.pdf]

This is a Multimedia Appendix to a full manuscript published in the J Med Internet Res. For full copyright and citation information see https://publichealth.jmir.org/2022/1/e32680/.

PILOT INTERVIEW QUESTIONS – TIM EVALUATION

Verbal introduction: The TIM Team is looking to make concrete improvements to the TIM system by gaining insight into how TIM is working, or has worked, for you and your agency. Specifically, we would like to understand how use of TIM has worked as a tool for symptom monitoring during the COVID-19 outbreak.

Before we get started, I would like to tell you about the procedures for this interview. Your participation is completely voluntary. If you want to stop at any time during the interview, please stop me, let me know, and I will pause or end the interview based on your preference. Additionally, we can skip over any questions that you prefer not to answer. Neither your decision to participate nor any of the specific feedback you provide will affect your access to TIM or the user support CDC provides. All of the reporting of the findings will be aggregated and not attributed to any individuals, agencies or jurisdictions

The interview is expected to take approximately **30-45 minutes**. In order to collect your responses, I will be taking notes throughout our call. Any references to personal identifiers during the interview will be omitted as I am taking notes or immediately following our discussion. All documentation relating to this interview will be saved on a secured and protected network. Your name will not be associated with these responses, and reports will use aggregate findings only.

Do you have any questions before we begin?

Thank you.

Section A. First we'd like to ask you about your agency affiliation and role in using TIM.

A1. With what type of agency are you affiliated? For example, federal, state, tribal etc.
A2. What is the name of your agency?
A3. In which State, U.S. Territory, IHS Service Area, or Tribal Nation is your agency located?
A4. In which month did you start using TIM?
A5. Is your agency still using TIM? Y/N **[If yes or I don't know, skip to question A7]**
A6. Why did your agency stop using TIM?
A7. I'm now going to read off a short list of TIM features. For each, please answer Yes or No if this TIM feature is something you manage for your agency's symptom monitoring efforts?:

- Campaign administration (e.g. setting up campaigns) Y/N
- Participant administration (e.g. adding or removing participants from campaigns) Y/N
- Alert administration (e.g. monitoring symptom, non-response, and opt-out alerts) Y/N
- User administration (e.g. adding new user accounts, sub-agencies)
- Data and reporting (e.g. generating participant, campaign, or alert reports, monitoring the dashboard)
- None of the above: what is your role with TIM?

A8. Do you serve as the primary or secondary Point of Contact for your agency's TIM team? These are Admin Users who serve as the main liaison between the CDC support team and their agency. This was established when you or your agency created a TIM account with the CDC TIM team. Y/N

Section B. This next set of questions will be about how you became aware of and interested in using TIM.

B1. How did you find out about TIM?
B2. What did you hear about TIM that compelled you to start using it?
B3. What, if anything, concerned you about using TIM?

Section C. Next we would like to better understand how you or your agency has used TIM.

C1. How has your agency used TIM? For example, has it been used to help with contact tracing, or monitor staff for short deployments? To conduct ongoing symptom monitoring for populations at risk to COVID-19? Please describe all purposes.

C2. Who have you or your agency monitored with TIM? For example, travelers, students, employees/deployed staff, cases, contacts. This answer may be the same as your answer to the last question – feel free to let me know if it is.

C3. What motivated this monitoring? For example, confirmed or possible exposures to a person with COVID-19? Proactive monitoring of staff? Mandated by a worksite SOP, policy, or directive? Voluntary precaution? Other reason?

C4. What other tools or systems have you or your agency used alongside or in conjunction with TIM?

C5. How have you or your agency used the TIM dashboard? For example, sharing data with your management.

C6. How have you or your agency used the Participant Activity Report generated by TIM? For example, changing workplace quarantine policies based on data? Please describe.

C7. How have you or your agency used the Campaign Summary Reports generated by TIM?

C8. How have you or your agency used the Participant Download extracts generated by TIM?

C9. How have you or your agency used the Responses download extracts generated by TIM?

C10. How have you or your agency used the data presented in the Alerts table? This is the table displayed after clicking the icon in the Alerts column of the Campaign Administration tab.

Section D. Next we would like to hear how TIM compares to other, potential options for conducting symptom monitoring.

D1. Were you conducting symptom monitoring prior to using TIM? Y/N **[If No, skip to question D13]**

D2. How were you conducting symptom monitoring before TIM?

When thinking back to symptom monitoring prior to TIM, we'd like to know, specifically, how the following things were different. We understand that the scale of your monitoring efforts may have changed since then. Please try to respond to the questions considering the effort, or burden you encountered per monitoring need with your previously used monitoring system or tool.

D3. Were you using more, less, or the same types of equipment or software applications?

D4. Were your costs to use that equipment or software more, less, or the same?

D5. Did you need more, less, or the same number of staff to conduct symptom monitoring?

D6. Did you spend more, less, or the same number of hours to conduct symptom monitoring?

Additionally, when thinking about the scale and quality of symptom monitoring with TIM…

D7. Is the number of people you are able to monitor higher, lower, or the same as it was before using TIM?

D8. Is the accuracy of the data collected on symptoms higher, lower, or the same?

D9. Is the completeness of the data collected on symptoms better, worse, or the same as it was before using TIM?

D10. Is the timeliness of the data collected on symptoms better, worse, or the same?

D11. Is TIM's ability to monitor diverse populations (in terms of sociodemographic, cultural, and linguistic backgrounds) better, worse, or the same, when compared to previously used symptom monitoring tools?

D12. Overall, how would you rate TIM for symptom monitoring compared previously used symptom monitoring tools? Better, worse, or the same? Please explain. **[Those who answer this question will then skip to Section E.]**

D13. If you had not been able to use TIM, how would you have conducted symptom monitoring? **[Those answering D13 will answer the remaining questions in section D. These questions are for respondents who answer No to question D1.]**

When thinking about how you might have conducted symptom monitoring without TIM, we'd like to know how the following things might have been different.

D14. Would you have needed more, less, or the same number of staff to conduct symptom monitoring?

D15. Would you have spent more, less, or the same number of hours to conduct symptom monitoring?

D16. Would you have used more, less, or the same types of equipment or software?

D17. Would your costs to use that equipment or software have been more, less, or the same?

Additionally, when thinking about the scale and quality of symptom monitoring with TIM…

D18. Would the number of people you would have been able to monitor been higher, lower, or the same without TIM?

D19. Would the accuracy of the data collected on symptoms have been higher, lower, or the same without TIM?

D20. Would the completeness of the data collected on symptoms have been better, worse, or the same without TIM?

D21. Would the timeliness of the data collected on symptoms have been higher, lower or the same

Section E. Now we would like to learn more about participants reporting symptoms and confirmed cases of COVID-19 identified through TIM.

E1. Through TIM, did you or your agency identify any participants who developed COVID-19 symptoms? Y/N **[If no, skip to section F.]**
E2. Do you think TIM enables more timely identification of symptomatic participants?

- E3. Please explain.

E4. Once the system notifies you of a symptomatic participant, do you have any role in or receive updates on participant follow-up? Y/N **[If no, skip to section F.]**
E5. Did you or others involved in participant follow-up identify any confirmed cases of SARS-CoV-2 infection from participants reporting symptoms to TIM? Y/N **[If no, skip to question F.]**
E6. Among your monitored population(s), what % were identified as cases?

- Date range
- Is this an exact and verified percentage? Y/N

E7. Do you think TIM enables more timely identification of those confirmed case(s) than had you used a different symptom monitoring method or tool? Y/N
E8. Why or why not?

Section F. This is the last section. We'd like to learn more about your experience onboarding for TIM and receiving support from CDC's TIM team.
F1. I'm now going to read off a short list of onboarding materials and activities. For each, please answer Yes or No if this is something you ever personally received from CDC.

- Initial/welcome emails Y/N
- TIM demos Y/N
- TIM User Guide Y/N
- TIM FAQs Y/N
- TIM Overview slides Y/N
- What other, if any, materials or onboarding assistance did you receive from CDC? **[If no onboarding materials or assistance received, skip to F7.]**

How helpful were each of the following instructions and guidance materials as it relates to onboarding yourself or others to TIM?

F2. Initial/welcome emails
F3. TIM demos
F4. TIM User Guide
F5. TIM FAQs
F6. TIM Overview slides
F7. What, if any, technical issues or other challenges did you experience that significantly affected your ability to manage campaigns in TIM?
F8. What, if any, technical issues or other challenges did you experience that significantly affected your ability to manage symptom alerts in TIM?

F9. What, if any, technical issues or other challenges did you experience that significantly affected your ability to manage non-response alerts in TIM?

F10. What, if any, technical issues or other challenges did you experience that significantly affected your ability to manage participants in TIM?

F11. What, if any, technical issues or other challenges did you experience that significantly affected your ability to manage users in TIM?

F12. Please describe any other technical issues or challenges that significantly affected your ability to use TIM for your agency's symptom monitoring activities.

F13. Since onboarding, which changes to the system made it work better for you?

F14. Please explain.

F15. Which, if any, system changes made it more difficult for you to use TIM?

F16. Please explain.

Did you submit any requests for technical support from the TIM team (either to the CDC mailbox or the TIM2 HelpDesk address)? Y/N **[If no, skip to question F22.]**

How would you rate your satisfaction with the TIM Team's responses to your technical support requests in terms of...

F18. Timeliness?

F19. Communication?

F20. Extent to which issues were Resolved

F21. Other relevant technical support qualities?

F22. Overall, how strongly would you recommend TIM for managing symptom monitoring activities?

F23. How long does your agency plan to use TIM? **[Only those who answer Yes to question A5 get asked this.]**

F24. Is there anything you would like to tell us about your use of TIM that we have not asked about? If so, please feel free to share.
