# Supplementary material for: An Evaluation of the Text Illness Monitoring (TIM) Platform for COVID-19: Cross-sectional Online Survey of Public Health Users
Source: JMIR Public Health Surveill. 2022 Feb 7;8(2):e32680. doi: 10.2196/32680 (PMC8823610; doi:10.2196/32680)
Supplement: Multimedia Appendix 2 [file publichealth_v8i2e32680_app2.pdf]

This is a Multimedia Appendix to a full manuscript published in the J Med Internet Res. For full copyright and citation information see https://publichealth.jmir.org/2022/1/e32680/.


# User Experience and Satisfaction Survey for CDC's Text Illness Monitoring (TIM)

The CDC Text Illness Monitoring (TIM) Team wants to understand how TIM has met your agency's needs during the COVID-19 pandemic. Your answers will be used to inform decisions and make improvements to the system and supporting materials. This survey is voluntary. Neither your decision to participate nor any of the specific feedback you provide will affect your access to TIM or the user support CDC provides. When we report the findings, data will be aggregated and not attributed to any individuals, agencies or jurisdictions. We estimate that it will take about 15 minutes to complete the survey. Importantly, you cannot save your responses and return to the survey; please plan to complete it in one session. Multiple respondents from an agency or jurisdiction are invited to participate. If you have questions about TIM please contact [redacted] If you have questions about this survey, please contact [redacted]

## A. Agency Affiliation and Role

A1. What best describes your agency type?

- ◯ Federal
- ◯ State
- ◯ Local
- ◯ Tribal
- ◯ Territorial
- ◯ Other______

A2. What is the name of your agency?

__________________________________

A3. In which state or U.S. Territory are you located?

__________________________________

A4. When did you start using TIM? (MM/YYYY) If unsure, please provide your best guess.

__________________________________

A5. Is your agency still using TIM?

- ◯ Yes
- ◯ No
- ◯ I don't know

A6. Why did your agency stop using TIM?

- ☐ No longer needed to monitor because the outbreak resolved
- ☐ Did not want to continue using a text-based system
- ☐ Other

Please specify.

__________________________________________

A7. When did you stop using TIM? (MM/YYYY) If unsure, please provide your best guess.

__________________________________

A8. Which of the following TIM features do you personally manage for your agency's symptom monitoring? (Check all that apply)

- ☐ Campaign administration (e.g. setting up campaigns)
- ☐ Participant administration (e.g. adding or removing participants to/from campaigns)
- ☐ Alert administration (e.g. monitoring symptom, non-response, and opt-out alerts)
- ☐ User administration (e.g. adding new user accounts, sub-agencies)
- ☐ Data and reporting (e.g. generating participant, campaign, and/or alert reports, monitoring the dashboard)
- ☐ None of the above (I helped get my agency set-up in TIM, but do not use the system)

A9. Do you serve as the primary or secondary Point of Contact for your agency's TIM team? These are Admin Users who serve as the main liaison between the CDC support team and their agency. This was established when you or your agency created a TIM account with the CDC TIM team.

- ◯ Yes
- ◯ No

## B. Decision to Use TIM

B1. How did you first find out about TIM? (Check all that apply)

- ☐ Outreach from CDC
- ☐ Leadership within agency
- ☐ TIM admin user
- ☐ Partner organization
- ☐ Word of mouth
- ☐ Other______

B2. What did you or your agency hear about TIM that made you interested in using it (Check all that apply)

- ☐ Better alternative to screening in person
- ☐ Better alternative to screening via phone
- ☐ Created and maintained by CDC
- ☐ No cost
- ☐ Could monitor large numbers of people
- ☐ Other______

B3. What, if anything, concerned you about using TIM? (Check all that apply)

- ☐ Privacy or security of health data
- ☐ Availability or reliability of cell phones
- ☐ Availability or reliability of cell service
- ☐ Time or staff burden to use it
- ☐ Timing of texts
- ☐ Charges for text messages
- ☐ None
- ☐ Other______

## C. Use of TIM

C1. How has your agency used TIM? (Check all that apply)

- ☐ Among staff, monitoring for development of symptoms
- ☐ Among staff, monitoring cases for worsening of symptoms
- ☐ Among staff, monitoring contacts of cases for development of symptoms
- ☐ Among community members, monitoring contacts of cases for development of symptoms
- ☐ Among community members, monitoring cases for development of symptoms
- ☐ Among community members, monitoring cases for worsening of symptoms
- ☐ Other______

| | |
|---|---|
| C2. What other tools or systems have you or your agency used alongside or in conjunction with TIM? (Check all that apply) | ☐ Agency or personal phones<br>☐ Contact tracing software______<br>☐ Data analysis software______<br>☐ In person screening<br>☐ Pen and paper<br>☐ Spreadsheets, such as Excel<br>☐ None<br>☐ Other______ |

How have you or your agency used the following TIM reports, extracts, and tables? If you have multiple campaigns, please think about how these functionalities were used to manage any of the campaigns.

| | |
|---|---|
| C3. How have you or your agency used the TIM dashboard? (Check all that apply) | ☐ Not using<br>☐ Reporting out<br>☐ Getting a sense of overall campaign/quick status check on responses<br>☐ Understanding completion and alert rates<br>☐ Other______ |
| C4. How have you or your agency used the Participant Activity Report? (Check all that apply) | ☐ Not using<br>☐ Confirming if messages are transmitted/Troubleshoot technical issues<br>☐ Adding to participants' electronic health record<br>☐ Catching outstanding alerts<br>☐ Confirming responses at specific times (e.g., before shifts start)<br>☐ Validation testing/internal review<br>☐ Initiate follow-up with participants<br>☐ Other______ |
| C5. How have you or your agency used the Campaign Summary Report? (Check all that apply) | ☐ Not using<br>☐ Reporting out<br>☐ Getting a sense of overall campaign/quick status check on responses<br>☐ Understanding completion and alert rates<br>☐ Other______ |
| C6. How have you or your agency used the Participant Download extract? (Check all that apply) | ☐ Not using<br>☐ Calculating completion and alert rates<br>☐ Moving people from one campaign to another<br>☐ Tracking TIM enrollment/completion<br>☐ Other______ |
| C7. How have you or your agency used the Responses download extract? (Check all that apply) | ☐ Not using<br>☐ Confirming if messages are transmitted/Troubleshoot technical issues<br>☐ Catching outstanding alerts<br>☐ Validation testing or internal review<br>☐ Initiate follow-up with participants<br>☐ Other______ |
| C8. How have you or your agency used the Alerts table? (Check all that apply) This is the table displayed after clicking the icon in the Alerts column of the Campaign Administration tab. | ☐ Not using<br>☐ Troubleshoot technical issues<br>☐ Catching outstanding alerts<br>☐ Initiate follow-up with participants<br>☐ Other______ |

## D. Other Options for Symptom Monitoring

D1. Were you conducting symptom monitoring prior to using TIM?

- ◯ Yes
- ◯ No

D2. How were you conducting symptom monitoring before TIM? (Check all that apply)

If you were monitoring more than one population (e.g. staff and community contacts), please select the method(s) used to monitor the largest of those populations.

- ☐ In person screening
- ☐ Contact tracing software______
- ☐ Other symptom monitoring application______
- ☐ Other______

**When thinking back to the symptom monitoring method you provided in response to Question D2, how were the following things different? We understand that the scale of your monitoring efforts may have changed since then. Please try to respond to the questions considering the effort or burden you encountered with your previously used monitoring system or tool.**

| | A lot more | A little more | The same | A little less | Much less | I don't know |
|---|---|---|---|---|---|---|
| D3. Compared to your previous system, is TIM more, less or the same in terms of your costs? | ◯ | ◯ | ◯ | ◯ | ◯ | ◯ |
| D4. Compared to your previous system, does TIM require more, less, or the same number of staff? | ◯ | ◯ | ◯ | ◯ | ◯ | ◯ |
| D5. Compared to your previous system, does TIM require more, less, or the same number of hours to conduct symptom monitoring? | ◯ | ◯ | ◯ | ◯ | ◯ | ◯ |
| D6. Compared to your previous system, is TIM more, less, or the same in effort for enrolling participants? | ◯ | ◯ | ◯ | ◯ | ◯ | ◯ |

**When thinking about the scale and quality of symptom monitoring with TIM...**

| | Much higher | A little higher | The same | A little lower | Much lower | I don't know |
|---|---|---|---|---|---|---|
| D7. Compared to your previous system, does TIM allow you to monitor a higher, lower, or comparable number of participants? | ◯ | ◯ | ◯ | ◯ | ◯ | ◯ |
| D8. Compared to your previous system, is the accuracy of the data collected on symptoms with TIM higher, lower, or the same? | ◯ | ◯ | ◯ | ◯ | ◯ | ◯ |

| | Much better | Somewhat better | The same | Somewhat worse | Much worse | I don't know |
|---|---|---|---|---|---|---|
| D9. Compared to your previous system, is the completeness of the data collected on symptoms with TIM better, worse, or the same? | ◯ | ◯ | ◯ | ◯ | ◯ | ◯ |
| D10. Compared to your previous system, is the timeliness of the data collected on symptoms with TIM better, worse, or the same? | ◯ | ◯ | ◯ | ◯ | ◯ | ◯ |

D11. Compared to your previous system, is your ability to monitor diverse populations (in terms of sociodemographic, cultural, and linguistic backgrounds) better, worse, or the same?

- ◯ Much better
- ◯ A little better
- ◯ Comparable/the same
- ◯ A little worse
- ◯ Much worse
- ◯ I don't know

D12. If you had not been able to use TIM, how would you have conducted symptom monitoring? (Check all that apply)

- ☐ Would have continued using the previous system
- ☐ Would have hired more staff
- ☐ Would have added phone calls
- ☐ Would have added in-person screening
- ☐ Would have added contact tracing software______
- ☐ Would have added another symptom monitoring application______
- ☐ I don't know
- ☐ Other______

D13. Reflecting on your overall experience with TIM, how would you rate it for symptom monitoring compared to previously used symptom monitoring system?

- ◯ Much better
- ◯ A little better
- ◯ Comparable/the same
- ◯ A little worse
- ◯ Much worse
- ◯ I don't know

D14. If you had not been able to use TIM, how would you have conducted symptom monitoring? (Check all that apply)

- ☐ Would have continued using the previous system
- ☐ Would have hired more staff
- ☐ Would have added phone calls
- ☐ Would have added in-person screening
- ☐ Would have added contact tracing software______
- ☐ Would have added another symptom monitoring application______
- ☐ I don't know
- ☐ Other______

**When thinking about how you might have conducted symptom monitoring without TIM, we'd like to know how the following things might have been different.**

| | Much more | A little more | The same | A little less | Much less | I don't know |
|---|---|---|---|---|---|---|

| | | | | | | |
|---|---|---|---|---|---|---|
| D15. Compared to this alternative system, would you have needed more, less, or the same number of staff to conduct symptom monitoring with TIM? | ○ | ○ | ○ | ○ | ○ | ○ |
| D16. Compared to this alternative system, would you have spent more, less, or the same number of hours to conduct symptom monitoring with TIM? | ○ | ○ | ○ | ○ | ○ | ○ |
| D17. Compared to this alternative system, would your costs have been more, less, or the same with TIM? | ○ | ○ | ○ | ○ | ○ | ○ |
| D18. Compared to this alternative system, would your effort to enroll participants be more, less or the same with TIM? | ○ | ○ | ○ | ○ | ○ | ○ |

**When thinking about the scale and quality of symptom monitoring with TIM...**

| | Much higher | A little higher | The same | A little lower | Much lower | I don't know |
|---|---|---|---|---|---|---|
| D19. Compared to this alternative system, would the number of people you would have been able to monitor been higher, lower, or the same with TIM? | ○ | ○ | ○ | ○ | ○ | ○ |
| D20. Compared to this alternative system, would the accuracy of the data collected on symptoms have been higher, lower, or the same with TIM? | ○ | ○ | ○ | ○ | ○ | ○ |

| | Much better | Somewhat better | The same | Somewhat worse | Much worse | I don't know |
|---|---|---|---|---|---|---|
| D21. Compared to this alternative system, would the completeness of the data collected on symptoms have been better, worse, or the same with TIM? | ○ | ○ | ○ | ○ | ○ | ○ |

D22. Compared to this alternative system, would the timeliness of the data collected on symptoms have been higher, lower or the same with TIM? ◯ ◯ ◯ ◯ ◯ ◯

## E. Symptom Follow-up

E1. Through TIM, did you or your agency identify any participants who developed COVID-19 symptoms? ◯ Yes ◯ No

E2. To what degree do you think TIM enables timely identification of symptomatic participants? ◯ Not at all ◯ Somewhat ◯ A lot ◯ Very much so

E3. Please expand on your answer. __________________________________

E4. Did a TIM campaign managed by your agency identify any symptomatic participants who were later confirmed to have SARS-COV2 infection?
◯ Yes
◯ No
◯ I don't know

E5. In how many different campaigns were cases identified?
◯ 1
◯ 2
◯ 3
◯ 4 or more
◯ I don't know

E6. Among Campaign One's monitored population(s), what percent were identified as confirmed COVID-19 cases?

a. Percent __________________________________
(If possible, please provide a verified and precise percent. If you only have an unverified estimate, that is also acceptable. Otherwise, you may leave this and fields b-c blank.)

b. Date range for the reported percent __________________________________
(MM/DD/YYYY - MM/DD/YYYY)

c. Is this an exact and verified percentage? ◯ Yes ◯ No

E7. Among Campaign Two's monitored population(s), what percent were identified as confirmed COVID-19 cases?

a. Percent __________________________________
(If possible, please provide a verified and precise percent. If you only have an unverified estimate, that is also acceptable. Otherwise, you may leave this and fields b-c blank.)

b. Date range for the reported percent __________________________________
(MM/DD/YYYY - MM/DD/YYYY)

c. Is this an exact and verified percentage? ◯ Yes ◯ No

E8. Among Campaign Three's monitored population(s), what percent were identified as confirmed COVID-19 cases?

a. Percent

__________________________________
(If possible, please provide a verified and precise percent. If you only have an unverified estimate, that is also acceptable. Otherwise, you may leave this and fields b-c blank.)

b. Date range for the reported percent

__________________________________
(MM/DD/YYYY - MM/DD/YYYY)

c. Is this an exact and verified percentage? ◯ Yes ◯ No

## F. Onboarding, Technical Assistance & Concluding Questions

F1. Have you personally received any of the following TIM onboarding or training materials from CDC? (Check all that apply)

- ☐ Initial/welcome emails
- ☐ TIM demos
- ☐ TIM User Guide
- ☐ TIM FAQs
- ☐ TIM Overview slides
- ☐ I did not receive any TIM onboarding or training materials from CDC

## How helpful were the following instructions and guidance related to onboarding yourself and/or others to TIM?

| | Very helpful | Somewhat helpful | Not that helpful | Not at all helpful |
|---|---|---|---|---|
| F2. Initial/welcome emails | ◯ | ◯ | ◯ | ◯ |
| F3. TIM demos | ◯ | ◯ | ◯ | ◯ |
| F4. TIM User Guide | ◯ | ◯ | ◯ | ◯ |
| F5. TIM FAQs | ◯ | ◯ | ◯ | ◯ |
| F6. TIM Overview slides | ◯ | ◯ | ◯ | ◯ |

F7. What, if any, technical issues or other challenges signficantly affected your ability to manage campaigns?

__________________________________

F8. What, if any, technical issues or other challenges significantly affected your ability to manage symptom alerts?

__________________________________

F9. What, if any, technical issues or other challenges significantly affected your ability to manage non-response alerts?

__________________________________

F10. What, if any, technical issues or other challenges significantly affected your ability to manage participants?

__________________________________

F11. What, if any, technical issues or other challenges significantly affected your ability to manage users in TIM?

__________________________________

F12. Please describe any other technical issues or challenges that significantly affected your ability to use TIM.

__________________________________

F13. Since onboarding, have there been any CDC-issued system changes to TIM?

- ◯ Yes
- ◯ No
- ◯ I don't know

F14. Please describe which changes made it easier to use TIM.

If none, please write "None."

__________________________________

F15. Please describe which changes made it more difficult to use TIM.

If none, please write "None."

__________________________________

F16. Did you submit any requests for technical support from the TIM team (either to the CDC mailbox or the TIM2 HelpDesk address)?

- ◯ Yes
- ◯ No

**How would you rate your satisfaction with the TIM Team's responses to your technical support requests in terms of...**

| | Very dissatisfied | Dissatisfied | Satisfied | Very satisfied | N/A |
|---|---|---|---|---|---|
| F17. Timeliness | ◯ | ◯ | ◯ | ◯ | ◯ |
| F18. Communication | ◯ | ◯ | ◯ | ◯ | ◯ |
| F19. Extent to which issues were resolved | ◯ | ◯ | ◯ | ◯ | ◯ |

F20. Overall, how strongly would you recommend TIM for managing symptom monitoring activities?

- ◯ Highly recommend
- ◯ Somewhat recommend
- ◯ Neither recommend nor discourage
- ◯ Somewhat discourage
- ◯ Highly discourage
- ◯ Unsure

F21. How long does your agency plan to use TIM?

__________________________________
(E.g., Until the pandemic ends, until we find a better alternative, I don't know)

F22. Do you have any concerns or foresee any challenges with using TIM in the near- or long-term? Please share with us.

__________________________________

F23. Are there any additional (i.e., non-COVID related) uses of TIM your agency has discussed or is considering? Please share with us.

__________________________________

F24. Is there anything else you would like to tell us about your use of TIM? Please share with us.

______________________________

You have finished the survey. Please click the SUBMIT button. Thank you for your time!
